# Effects of low-load blood flow restriction vs. high-load resistance training on upper-body strength in male collegiate gymnasts: A randomized controlled trial

**DOI:** 10.1016/j.jesf.2026.200456

**Published:** 2026-02-05

**Authors:** Aojie Li, Jing Tang, Kaiqi Zheng, Jingyi Chen, Guangshun Wang, Daoguang Feng

**Affiliations:** a School of Physical Education and Sports Science, South China Normal University, Guangzhou, 510006, China; b Department of Physical Education and Sports Research, South China Agricultural University, Guangzhou, 510642, China

**Keywords:** Blood flow restriction, Resistance training, Gymnastics, Upper-body strength, Maximal strength, Strength endurance

## Abstract

**Purpose:**

High-load resistance training (HRT) is the standard for developing strength, characterized by high mechanical loads. Low-load training with blood flow restriction (BFR-LRT) has emerged as an alternative that uses lower mechanical loads but greater repetition volume to induce metabolic stress. While these training modalities impose differing physiological demands, the extent to which they produce comparable adaptations in highly trained athletes remains unclear. This study aimed to compare the effects of a 6-week BFR-LRT program versus a traditional HRT program on upper-body maximal strength and strength endurance in male collegiate gymnasts.

**Methods:**

Thirty male collegiate gymnasts completed the experiment in three parallel groups: HRT (3 sets of 10 repetitions at 75% 1-repetition maximum [1RM]), BFR-LRT (1 set of 30 and 3 sets of 15 repetitions at 30% 1RM), or a control group (CG) that performed only regular gymnastics training. The 6-week intervention was preceded by familiarization and reliability testing. Upper-body maximal strength (1RM for bar dip, pull-up, and shoulder press) and strength endurance (maximal repetitions for bar dips 60s, pull-ups 40s, and handstand push-ups 40s) were assessed at baseline and post-intervention. A one-way Analysis of Covariance (ANCOVA), with baseline scores as a covariate, was used to compare post-intervention outcomes. To formally interpret non-significant comparisons between the HRT and BFR-LRT groups, a follow-up Bayesian ANCOVA was performed. Additionally, mediation analysis was conducted to determine if improvements in maximal strength mediated the observed gains in strength endurance.

**Results:**

Both HRT and BFR-LRT produced significantly greater improvements than the control group across all outcomes. The primary comparison revealed no statistically significant differences between the HRT and BFR-LRT groups on any measure. The 95% confidence intervals for the adjusted mean differences consistently included zero, supporting their comparable efficacy. Follow-up Bayesian analysis consistently provided anecdotal evidence supporting comparable efficacy between the two training groups (Bayes Factor BF_01_ > 2.0 for five of six outcomes). Exploratory mediation analysis revealed divergent mechanistic pathways for these adaptations: for handstand push-ups, endurance gains were statistically mediated by increased maximal strength in the HRT group, whereas this pathway was not significant for the BFR-LRT group.

**Conclusion:**

In this cohort of elite gymnasts, a 6-week BFR-LRT program produced comparable adaptations in upper-body strength and strength endurance to traditional HRT, with Bayesian analysis supporting their similar efficacy. Mediation analysis revealed that the pathways to these gains were modality-specific: improvements in handstand push-up endurance were statistically mediated by gains in maximal strength for the HRT group, but not for the BFR-LRT group. These findings establish BFR-LRT as a viable low-load training alternative that may stimulate adaptations through distinct physiological mechanisms, offering important practical implications for managing training volume and mitigating joint stress.

## 1 Introduction

Resistance training represents a cornerstone of athletic preparation across numerous sports, with particular importance in disciplines requiring high levels of muscular strength and power.^1^^,^^2^ Traditional high-load resistance training (HRT), typically performed at loads exceeding 70% of one-repetition maximum (1RM), has long been considered the gold standard for developing maximal strength and muscle hypertrophy.^3^ However, the demanding nature of high-load training presents several challenges for athletes, particularly those in sports requiring high training volumes and frequent competitions. These limitations include increased risk of overuse injuries, prolonged recovery requirements, and potential interference with sport-specific technical training^4^, ^5^, ^6^. Male artistic gymnastics exemplifies such a sport, where athletes must develop exceptional upper-body strength while maintaining the capacity for high-volume skill training across multiple apparatus.^7^^,^^8^ In recent decades, there has been a growing interest in alternative training methodologies. Among these, blood flow restriction (BFR) training has emerged as a particularly promising approach. Research has demonstrated that augmenting low-load resistance training with blood flow restriction to the active musculature can produce significant hypertrophy and strength gains using loads as low as 30% of one repetition maximum (1RM)^9^, ^10^, ^11^. BFR involves the application of external pressure to the proximal portion of exercising limbs, creating a state of partial vascular occlusion that allows arterial inflow while restricting venous return.^12^

Traditional high-load resistance training (HRT) is characterized by high mechanical tension, which is proposed to stimulate strength and muscle hypertrophy through pathways involving neuromuscular and protein synthetic responses. In contrast, blood flow restriction with low-load training (BFR-LRT) is characterized by minimal mechanical tension but generates profound metabolic stress^13^, ^14^, ^15^. This metabolic environment is thought to provide an alternative stimulus for adaptation, potentially by enhancing the recruitment of high-threshold motor units despite the low external load. However, the precise mechanisms linking these distinct training characteristics to functional strength gains remain incompletely understood, and the assumption of causality is not yet fully supported by empirical data^16^, ^17^, ^18^. This uncertainty is particularly pertinent in highly trained athletes, who may exhibit distinct adaptive responses compared with untrained individuals. Accordingly, the present study aims to compare the practical outcomes of these two training modalities. However, a review of the current literature reveals notable gaps in our understanding of BFR applications. Firstly, although many studies have investigated the effects of BFR, this research has mostly focused on the lower body or single-joint upper body exercises (e.g. biceps curls and triceps extensions).^11^^,^^14^^,^^15^^,^^19^^,^^20^ In contrast, the effects of BFR during multi-joint, compound upper-body movements, such as pull-ups and shoulder presses, which are central to gymnastics, remain relatively under-investigated. Second, the majority of BFR research has focused on untrained or recreationally active populations.^21^^,^^22^ The extent to which BFR-induced adaptations translate to highly trained athletes remains unclear, particularly in sports where upper-body strength represents a primary performance determinant. Training status appears to moderate BFR responses, with some evidence suggesting that trained individuals may derive greater benefits from BFR compared to high-load training than their untrained counterparts.^21^

Male artistic gymnastics presents unique physiological demands that make it an ideal model for investigating upper-body training interventions.^7^^,^^8^ Success in gymnastics requires exceptional relative strength, particularly in the upper body, where athletes must support and manipulate their body weight through complex movement patterns.^7^^,^^23^ Events such as still rings, parallel bars, and high bar demand sustained isometric strength, dynamic strength, and strength endurance.^7^^,^^24^ The training characteristics of competitive gymnasts create additional considerations for strength development programs. Gymnasts typically train 20-30 h per week, with the majority of time dedicated to skill acquisition and routine practice.^25^^,^^26^ This high-volume technical training often limits the time and recovery capacity available for supplementary strength training.^23^ Moreover, the high-impact nature of gymnastics training places considerable stress on the musculoskeletal system, making recovery optimization a critical concern.^7^ Research examining strength training in gymnasts has demonstrated the importance of upper-body strength for performance.^8^^,^^23^ However, most gymnastics-specific strength research has focused on traditional high-load methods or sport-specific exercises, with limited investigation of alternative training modalities such as BFR.

Based on the available evidence and theoretical considerations, we hypothesized that both HRT and BFR-LRT would produce significant improvements in upper-body maximal strength and strength endurance compared to a control condition in male collegiate gymnasts. Furthermore, we hypothesized that there would be no significant differences in training effects between the HRT and BFR-LRT groups, supporting the concept that BFR can serve as an effective alternative to traditional high-load training. The primary objective of this study was to compare the effects of a 6-week BFR-LRT intervention versus HRT on upper-body maximal strength and strength endurance in male collegiate gymnasts. This comparison aimed to determine if BFR-LRT is a viable alternative to traditional high-load training, which would provide a basis for its practical application in managing training load and mitigating joint stress in this elite athletic population.

## 2 Methods

### 2.1 Study design

This study employed a three-arm, parallel-group, randomized controlled design to compare the effects of a 6-week training intervention. Thirty male collegiate gymnasts were randomly allocated to one of three groups: high-load resistance training (HRT), blood flow restriction with low-load resistance training (BFR-LRT), or a control group (CG) that performed only regular gymnastics training.

The study protocol was approved by the South China Normal University Institutional Review Board. This research was conducted in accordance with the ethical principles for human investigation outlined in the Declaration of Helsinki, and all participants provided written informed consent before their enrollment in the trial. The trial was designed and reported by the Consolidated Standards of Reporting Trials (CONSORT) guidelines.^27^^,^^28^ The experimental timeline consisted of a familiarization session, baseline testing (Week 1), the 3-week intervention period, retest 1RM of the resistance training movements, the 3-week intervention period, and post-intervention testing (Week 8).

### 2.2 Participants

Participants were required to meet the following inclusion criteria: (1) male collegiate gymnasts aged 18-25 years; (2) minimum 3 years of competitive gymnastics experience; (3) engaged in at least 15 h per week of gymnastics-specific training at the time of enrollment; (4) no history of upper-body injury in the previous 6 months; (5) no use of performance-enhancing substances; and (6) provision of written informed consent. Exclusion criteria included: (1) cardiovascular disease or hypertension; (2) history of blood-clotting disorders; (3) current use of anticoagulant medications; (4) previous experience with BFR training; (5) concurrent participation in structured resistance training programs; and (6) inability to complete baseline testing procedures.

Thirty-five gymnasts were assessed for eligibility, each of whom was able to perform Level 6 of the Chinese Gymnastics Program Level Required Movements, with Level 6 being an improvement level, and each of whom had competed at the provincial level. Three athletes were excluded—two due to pre-study injuries and one because of test scheduling conflicts—leaving 32 eligible participants who provided written informed consent and initiated the trial.

Following the completion of all baseline testing procedures (Week 1), the 32 enrolled gymnasts were randomly allocated via a computer-generated sequence into one of three parallel groups: blood flow restriction with low-load training (BFR-LRT; n = 11), high-load resistance training (HRT; n = 11), and control (CG; n = 10). This randomization was performed immediately before the commencement of the 6-week intervention period. Allocation was concealed in opaque, sequentially numbered envelopes opened by a study coordinator not involved in outcome assessments. Each group adhered to its assigned protocol over a six-week intervention period, while continuing with its regular athletic training routines.

**Fig. 1:**
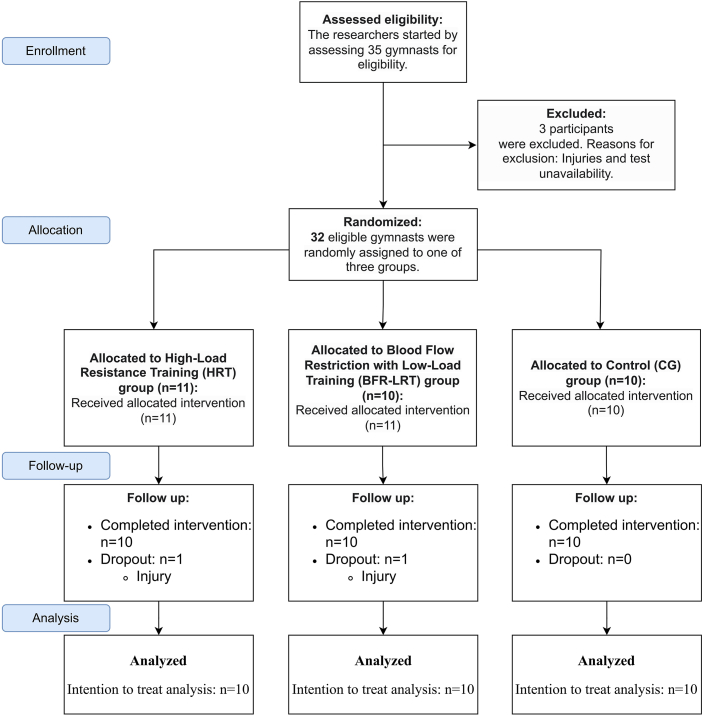
Consort flow diagram.

Follow-up assessments were completed by 10 participants in the BFR-LRT group (one withdrawal due to injury), 10 in the HRT group (one withdrawal due to injury), and all 10 in the CG group. Data from the 30 gymnasts who completed the intervention were analyzed according to the intention-to-treat principle (n = 10 per group), with incomplete cases removed from the analysis. The complete flow of participants through each stage of the trial is detailed in the CONSORT diagram (Fig. 1).

The baseline demographic and physical characteristics of the participants in each of the three groups are summarized in Table 1. As per the study design, the weekly training volume was intentionally higher for the HRT and BFR-LRT intervention groups than for the control group.

**Table 1:** Physical characteristics of CG、HRT, and BFR-LRT.

	CG (n = 10)	HRT (n = 10)	BFR-LRT (n = 10)
Age (yr)	20.1 ± 1.1	20.4 ± 1.2	20.6 ± 1.2
Height (cm)	171.0 ± 4.5	170.9 ± 4.7	171.6 ± 5.9
Body Mass (kg)	67.0 ± 5.7	66.2 ± 4.2	66.1 ± 5.7
Shoulder Width(cm)	46.8 ± 2.9	45.5 ± 3.4	45.8 ± 2.6
Training Years(yr)	5.3 ± 1.3	5.1 ± 1.1	5.1 ± 1.4
Systolic Blood Pressure(mmHg)	120.9 ± 7.4	123.2 ± 6.3	121.2 ± 7.1
Training Volume (h/week)	15	18	18

### 2.3 Training interventions

During the 6-week intervention phase, all participants performed flexibility as well as technical training 5 days per week. However, only the BFR-LRT and HRT groups also performed resistance intervention training 3 days per week. For the resistance intervention protocol, the focus was on resistance training movements related to gymnastics specialization, including pull-ups, double-bar arm extensions, and Smith barbell shoulder presses (see Table 2). The decision not to include inverted push-ups was based on the difficulty of quantifying the external load, a challenge that can be circumvented by opting for the barbell shoulder press, which exhibits a similar movement pattern to the inverted push-up. This approach enables the quantification of the external load. In order to select an appropriate training load, it is necessary to refer to the study conducted by Ozaki et al.^29^ in 2013. The training load and volume of the HRT group were set at 75% of the one-repetition maximum (1-RM) and 30 repetitions (3 sets of 10 repetitions each, with a 2-3 min rest period between sets). The training load and volume of the BFR-LRT group were set at 30% of the 1-RM and 75 repetitions, respectively (30 repetitions for set 1, followed by 3 sets of 15 repetitions each with 2-3 min of rest between sets). The training protocol was structured to equate the calculated total volume load between intervention groups, despite the large differences in external load per repetition (75% 1RM vs. 30% 1RM). Total volume load was calculated as sets × repetitions × %1RM, yielding an identical value of 22.5 × 1RM for both groups. While matching volume load is a common method for comparing training programs, we acknowledge that this calculation of external work does not necessarily imply equivalent physiological stress.^30^ Therefore, this design allowed for a pragmatic comparison of the two training modalities under conditions where the prescribed external volume was mathematically matched. In contrast, the control group (CG) did not undertake resistance training but only engaged in flexibility and technical exercises five times per week. The inclusion of a control group that maintains regular training serves a similar purpose to a time-matched, non-exercise control group, as it is essential for evaluating whether the observed effects are a direct result of the specific training intervention rather than other confounding factors.^31^ At the end of the third week of intervention, the 1RM of pull-ups, double-bar arm extensions, and barbell shoulder presses were reassessed in order to adjust the training load for subsequent training.

**Table 2:** 6-week, 3-times-weekly intervention protocols for HRT and BFR-LRT groups.

Groups	Exercise	Loads	Reps	Sets	Interval	Blood-flow pressure
HRT	Pull-up	75%1RM	10	3	2-3min	0
	Bar Dip	75%1RM	10	3	2-3min	0
	Shoulder Press	75%1RM	10	3	2-3min	0
BFR-LRT	Pull-up	30%1RM	30/15/15/15	4	2-3min	70%SBP (cuffs remained inflated)
	Bar Dip	30%1RM	30/15/15/15	4	2-3min	70%SBP (cuffs remained inflated)
	Shoulder Press	30%1RM	30/15/15/15	4	2-3min	70%SBP (cuffs remained inflated)
CG	Avoid any structured strength training during the study period.					

Shoulder Press, Smith Machine Shoulder Press; SBP, Systolic Blood Pressure.

All participants in both the HRT and BFR-LRT groups successfully completed all prescribed repetitions and sets during every training session throughout the 6-week intervention. Adherence to the prescribed training volume was prioritized to ensure a direct comparison of the two protocols. It was observed that in the final repetitions of some sets, participants in both groups occasionally exhibited minor body sway as a compensatory mechanism to overcome fatigue. While strict form was verbally encouraged by supervisors, completion of the prescribed repetition count was ensured. This behavior was noted with similar frequency in both the HRT and BFR-LRT groups, suggesting a comparable level of effort and fatigue was reached relative to their respective protocols.

### 2.4 Blood flow restriction pressure

Prior to each training session, participants in the BFR-LRT group rested quietly in a seated position for 10 min. Resting systolic blood pressure (SBP) was then measured on the non-dominant arm using a calibrated, automated blood pressure monitor (Xiaomi Mi Smart Upper Arm Blood Pressure Monitor, Model: XMTK10). The measurement was performed three times with a 1-min interval between each reading; the average of the final two readings was used to establish the SBP for that day's session. Baseline SBP was also recorded for all participants during the initial assessment week (Week 1). The mean baseline SBP values for each group are presented in Table 1.

The occlusion pressure for the BFR-LRT group was set at 70% of the individual's SBP. This relative pressure was selected because it has been shown to effectively restrict venous outflow while maintaining sufficient arterial inflow in the upper limbs, thereby inducing a significant metabolic and hypoxic stimulus for adaptation without complete vascular occlusion.^32^ For the intervention, an adjustable pneumatic blood flow restriction cuff (Theratool, China) was placed on the most proximal portion of the upper arm, distal to the deltoid and proximal to the biceps muscle. The cuff was inflated to the target pressure before the first set and remained inflated throughout all four sets of the exercise, including the 30-s rest intervals, to maximize metabolic accumulation.

### 2.5 Experimental procedures

**Fig. 2:**
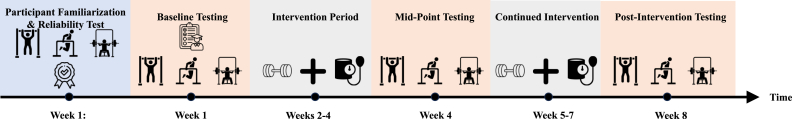
Experimental procedures timeline.

The study was conducted over an 8-week period, comprising a familiarization week, a baseline testing week, a 6-week intervention period with a mid-point assessment, and a final week for post-intervention testing. A schematic of the complete experimental timeline is provided in Fig. 2.

#### 2.5.1 Participant familiarization and reliability Test (Week 1)

All participants attended a single familiarization session at week 1. The purpose of this session was to thoroughly acquaint participants with the specific techniques and demands of all six performance tests (bar dip 1RM, pull-up 1RM, Smith machine shoulder press 1RM, and the three corresponding strength endurance tests). This was done under the direct supervision of the research team to standardize procedures and minimize any learning effects between subsequent testing sessions.

Following a 48-h rest period after the familiarization session, twenty participants performed the full battery of six performance tests. They repeated the same battery of tests 48 h later to complete the test-retest protocol. Data from these two sessions were used to establish the reliability of each measure, as detailed in the Outcome Measures section.

#### 2.5.2 Baseline and post-intervention testing (Week 1 and week 8)

All performance assessments (both baseline and post-intervention) were conducted under strictly controlled conditions. Sessions were scheduled at the same time of day (±2 h) for each participant, following a 12-h overnight fast and 24 h of abstinence from strenuous exercise and alcohol consumption. To standardize the testing protocol and minimize the influence of potential confounding variables such as residual fatigue, all performance assessments were conducted across two separate sessions, separated by 48 h. This protocol was implemented identically for all participants during both baseline and post-intervention testing, ensuring that any systematic effect of the testing procedure itself would be consistent across all assessments. Session 1 was dedicated to assessing maximal strength (1RM for bar dip, pull-up, and shoulder press). Session 2 was dedicated to assessing strength endurance (maximum repetitions for bar dip, pull-up, and handstand push-up). Anthropometric measurements as well as medical examination at baseline testing are performed during Session 1. Each testing session began with a standardized warm-up. Following the warm-up, the respective strength or endurance tests were performed in a fixed order, with a 5-min passive rest period between each assessment.

#### 2.5.3 Mid-intervention assessment (end of week 4)

At the end of the third week of the intervention period, participants in all three groups (HRT, BFR-LRT, and CG) underwent a re-assessment of their 1RM for the three resistance training movements (pull-ups, bar dip, and shoulder press). For the HRT and BFR-LRT groups, this assessment was used to adjust the training loads for the final three weeks of the intervention, ensuring the principle of progressive overload was maintained. Crucially, the control group also completed this mid-point 1RM test to control for any potential confounding training stimulus that might result from the repeated high-load testing itself. This ensures that any observed differences between the training groups and the control group can be more confidently attributed to the intervention rather than the testing protocol.

### 2.6 Outcome measures

#### 2.6.1 Reliability and variability of outcome measures

Given that the performance tests were specifically adapted for a highly trained cohort of collegiate gymnasts, a preliminary study was undertaken to evaluate their acute test–retest reliability prior to the main trial. This 48-h assessment served to confirm the reproducibility of the sport-specific metrics before their application in the intervention. Nonetheless, short-term reliability testing is insufficient to account for the cumulative measurement error that may arise over a 6-week intervention period. Accordingly, the inclusion of a time-matched non-exercise control group was deemed essential, as it provided a reference point for determining whether observed changes were attributable to the training intervention.^31^

The twenty individuals in this reliability sample were also participants in the main study. To address the potential confounding effect of this additional testing exposure, all reliability and baseline assessments were completed before the randomization process. By randomizing after all preliminary testing, these participants were allocated randomly across the high-load, low-load BFR, and control groups. This procedure ensures that any potential learning effect or training stimulus from the additional testing was not systematically biased toward any single group and was instead treated as random variance within each group. This is further addressed as a limitation in the discussion.

The statistical methods for this reliability analysis are detailed in Section 2.7. The analysis confirmed that all six outcome measures demonstrated good to excellent reliability (all ICCs >0.9) and acceptable measurement variability (all CVs <10%), thereby validating their use as primary outcome variables for this randomized controlled trial. The detailed reliability and variability data are presented in Table 3.

**Table 3:** Test-retest reliability and variability of outcome measures.

Outcome Measure	ICC (95% CI)	SEM	CV (%)
Maximal Strength			
Bar Dip 1RM (kg)	0.95 (0.89-0.98)	1.54 kg	1.64%
Pull-up 1RM (kg)	0.95 (0.89-0.98)	0.99 kg	1.24%
Smith Machine Shoulder Press 1RM (kg)	0.92 (0.81-0.97)	1.18 kg	1.50%
Strength Endurance			
Max Bar Dips (reps in 60s)	0.96 (0.89-0.98)	0.95reps	3.88%
Max Pull-ups (reps in 40s)	0.93 (0.82-0.97)	1.09reps	5.19%
Max Handstand Push-ups (reps in 40s)	0.91 (0.79-0.96)	0.65reps	5.32%

ICC = Intraclass Correlation Coefficient; CI = Confidence Interval; SEM = Standard Error of Measurement; CV = Coefficient of Variation.

#### 2.6.2 Maximal strength

##### 2.6.2.1 Bar dip 1RM

The subjects completed the movement on a standard double bar, which was uniformly 20 cm wider than shoulder width for arm flexion. After warm-up, the subject rested for 5 min before commencing the formal execution of the bar dips, utilizing a load equivalent to 80% of the subject's self-reported 1 RM. The elbow joint was maintained in the flexed position at 90° throughout the movement, and the elbow joint in the extended position was fully straightened. The exercise was considered successful if the subject completed it correctly. Following the conclusion of the task, a 5-min period of rest was recommended. The weight was increased by 2.5 to 10 kg (based on their self-reported difficulty of the preceding successful attempt), and arm flexion and extension were continued in a standardized movement until the subject was unable to straighten the elbow at the specific weight. The load prior to increasing the weight was recorded, and the maximum double-bar arm flexion strength/body weight (kg) was calculated. During arm flexion extension, the subjects were instructed to refrain from relying on their lower back or legs to lift the load, and to maintain a tight hip position, with the legs and toes kept in a closed position, and the knees either straight or uniformly bent, as appropriate, depending on the height of the double bar.

##### 2.6.2.2 Pull-up 1RM

The pull-ups were executed on a standard bar, with a grip that was 20 cm wider than shoulder-width. After warm-up, the pull-ups were performed at 80% of the subjects’ self-reported 1 RM. The elbows and shoulders were fully extended at the bottom, the chin was positioned over the bar at the top, and the head was held in a neutral position to prevent the chin from lifting. Following the completion of the pull-up set, the subject rested for a period of 5 min. Thereafter, the weight was increased by a margin of 2.5 to 10 kg. The magnitude of the increase was determined for each participant based on their self-reported difficulty of the preceding successful attempt, in consultation with the testing supervisor. This approach was used consistently to guide the progression to the one-repetition maximum. The pull-up exercise was performed in a standardized manner until the subject was unable to complete the movement with the selected weight. The load was recorded prior to any increase in weight, and the 1RM of the pull-up was documented. During the pull-up, the subject was required to refrain from relying on the waist or legs for strength and to maintain a tight grip, with the legs and toes kept in a closed position and the knees straight (or uniformly bent if the height of the bar was insufficient).

##### 2.6.2.3 Smith Machine Shoulder Press 1RM

The subjects performed barbell shoulder presses in a seated position on a 90° bench in a Smith rack, uniformly using a full grip width (1.5 × shoulder peak distance) and a positive grip for the barbell shoulder press. Following a warm-up consisting of wrist, elbow, and shoulder stretches, a push-up warm-up, and a shoulder press warm-up, the barbell shoulder press was formally begun with a load of 80% of the subjects' self-reported 1 RM. The completion of the movement was defined as the point at which the elbow joint was flexed to 90° at the bottom position and fully extended at the top position. Following this, a 5-min rest period was recommended. Subsequently, the weight was increased by increments of 2.5-10 kg, with the magnitude of each increase being guided by the participant's self-reported difficulty of the previous successful lift. The barbell shoulder press was performed in a standardized manner for each attempt until the participant was unable to complete the movement by fully straightening the elbow. The load of the final successful repetition was recorded, and this value was determined to be the 1RM for the seated barbell shoulder press.

#### 2.6.3 Upper-body strength endurance

The measures for upper-body strength endurance were chosen to reflect the specific demands of gymnastics. Rather than using traditional tests of muscular endurance (e.g., repetitions to failure at a percentage of 1RM), we adopted a battery of gymnastics-specific performance tests. The selection of these timed repetition protocols was based on the validated physical fitness evaluation standards for male collegiate gymnasts developed by Zheng et al. (2014).^33^ This work established these measures as reliable and ecologically valid assessments of the endurance capacities critical for gymnastics performance.

##### 2.6.3.1 Maximum bar dips repetitions in 60s

The subjects were instructed to perform the movements on a standard double bar, which was uniformly 20 cm wider than the width of the shoulders for arm flexion. Following a warm-up, a 5-min rest period was used to officially begin the test. The subjects were required to complete the test as expeditiously as possible, with the elbow joint in the bottom position flexed to 90° and the elbow joint in the top position fully extended. After the tester issued the “start” command, the subject initiated the test. One tester ensured that the subject met the standard for each arm flexion and recorded the number of successful repetitions, while another tester recorded the time and issued the commands. Any repetitions that did not meet the standard were not counted. During the process of arm flexion and extension, the subjects were required to refrain from relying on the waist or legs for assistance. The hips were kept tight, the legs together, the toes straight, and the knees straight (or uniformly bent if the height of the double bar was insufficient).

##### 2.6.3.2 Maximum pull-ups repetitions in 40s

Subjects completed the movement on a standard bar, uniformly using a positive grip that was 20 cm wider than shoulder width.^34^ The protocol for pull-ups was as follows: participants were required to perform a series of warm-up exercises, including wrist, elbow, and shoulder stretches, followed by hang warm-ups and self-weighted pull-ups. Following this preparatory phase, a 5-min rest period was allocated before commencing the formal test. Participants were required to execute one complete repetition as expeditiously as possible, with elbows and shoulders fully extended at the bottom position, chin positioned over the bar at the top position, and head maintained in a neutral position to prevent chin lifting. Following the tester's command to commence, the subject initiated the test. One tester was responsible for ensuring that the subject met the standard for each pull-up and recorded the number of pull-ups completed. The second tester's role was to record the time and to issue the command. It was important to note that pull-ups that did not meet the standard were not counted. During the pull-up process, the subject was required to ensure that they did not rely on the waist and legs to apply force. Additionally, they were expected to maintain a tight buttock position, keeping the legs together and the toes and knees straight. In the event that the height of the bar was insufficient, the knees were bent uniformly.

##### 2.6.3.3 Maximum handstand push-ups repetitions in 40s

Subjects performed push-ups on an inversion stand facing the wall, with the stand positioned at a uniform width of 20 cm wider than the subject's shoulders and 10 cm away from the wall. After a warm-up, the test began after a 5-min rest. Subjects were required to touch the head at the bottom position 15 cm from the ground sponge, at the top position elbow and shoulder joints were fully extended, and the movement was completed as quickly as possible. Throughout the entire test, subjects were allowed only to gently press their feet against the wall and were required not to collapse their waist or bend their legs. The test was supervised by two members of the research team. One tester was responsible for ensuring that each repetition met the required performance standard and for recording the total number of valid push-ups. A second tester was responsible for timing the test and issuing the necessary commands. Any push-up that did not meet the standard was not included in the participant's final count.

### 2.7 Statistical analysis

All descriptive data are presented as mean ± standard deviation (SD), unless otherwise specified. The normality of data distribution for all variables was assessed using the Shapiro-Wilk test, given the sample size.

Statistical analysis of reliability was performed as follows. Relative reliability was assessed using the Intraclass Correlation Coefficient (ICC). A two-way mixed-effects model with an absolute agreement definition was used, as this is the most appropriate model for test-retest designs.^35^ ICC values were interpreted as: <0.50 (poor), 0.50–0.75 (moderate), 0.75–0.90 (good), and >0.90 (excellent).^35^ To quantify absolute measurement error relative to the mean, the coefficient of variation for the standard error of measurement (CV_SEM_) was calculated, following the principles outlined by Atkinson and Nevill (1998).^36^ The formula used was: CV_SEM_ (%) = (SEM/Mean) × 100%, where the SEM represents absolute within-subject measurement error and the mean was derived from the first testing session.

The primary statistical analysis to compare post-intervention outcomes among the three groups (CG, BFR-LRT, HRT) was a one-way Analysis of Covariance (ANCOVA). This approach is considered the gold standard for analyzing pre-post randomized trials. By adjusting for the pre-intervention (baseline) value as a covariate, ANCOVA provides a more precise and powerful estimate of the treatment effect than an analysis of change scores, and it effectively controls for any chance baseline imbalances between the groups.^37^ For each of the six outcome measures, the post-intervention value served as the dependent variable, the group allocation (CG, BFR-LRT, HRT) served as the independent factor, and the corresponding pre-intervention (baseline) value was included as a covariate. The critical ANCOVA assumption of homogeneity of regression slopes was tested for each model by examining the significance of the group-by-covariate interaction term. As this interaction was found to be non-significant for all outcomes (p > 0.10), this assumption was met, validating the use of ANCOVA. When a significant main effect for the group factor was detected (p ≤ 0.05), post-hoc pairwise comparisons were conducted between the HRT, BFR-LRT, and CG groups, with specific differences reported as adjusted mean differences and their 95% confidence intervals (CIs). In line with recommendations for making inferences on individual hypotheses, an unadjusted alpha level of 0.05 was used for each pairwise comparison.^38^ The effect size for the overall ANCOVA model was reported as partial eta-squared (ηp^2^), providing an estimate of the proportion of variance in the dependent variable explained by the group factor after controlling for the covariate. Values for ηp^2^ were interpreted as small (0.01), medium (0.06), and large (0.14).^39^ To quantify the magnitude of change within each group from pre-to post-intervention, the standardized effect size was calculated as Cohen's d, using the standard deviation of the change score as the denominator (d = Mean_change_/SD_change_). This approach reflects the magnitude of the intervention effect relative to response variability(rather than sample population variability).^40^^,^^41^ Cohen's d values were interpreted as trivial (<0.2), small (0.2–0.5), medium (0.5–0.8), and large (>0.8).^42^ All statistical analyses were performed using IBM SPSS Statistics for Windows (Version 26.0; IBM Corp., Armonk, NY, USA). Figures were generated using GraphPad Prism (Version 6.0; GraphPad Software Inc., San Diego, CA, USA). Statistical significance for all inferential tests was accepted at an alpha level of p ≤ 0.05.

To provide a more informative interpretation of non-significant comparisons between the HRT and BFR-LRT groups, we supplemented our frequentist analyses with a Bayesian approach. For each outcome measure, a Bayesian ANCOVA was conducted using JASP software (Version 0.18.3.0), with post-intervention scores as the dependent variable, group allocation (HRT vs. BFR-LRT) as the fixed factor, and baseline scores as a covariate. We used the default Cauchy prior width of 0.707. We report the resulting Bayes Factor in favor of the null hypothesis (BF_01_), which quantifies the relative evidence for the hypothesis of no difference versus the hypothesis of a difference between the groups. A BF_01_ between 1 and 3 is interpreted as anecdotal evidence for the null hypothesis.

To formally test the hypothesis that improvements in maximal strength mediated the gains in strength endurance, and to investigate whether this mechanistic pathway differed between the two training modalities, we conducted three separate mediation analyses (one for each exercise: bar dip, pull-up, and handstand push-up) using the PROCESS macro (v5.0, Model 4) for SPSS.^43^ For these analyses, the independent variable (X) was a single, three-level categorical variable representing the group allocation (CG, HRT, BFR-LRT). This variable was specified as multicategorical within PROCESS, a procedure that allows for the estimation of relative indirect effects for each intervention group compared to a common reference group (the control group). The change score in maximal strength for the corresponding exercise served as the mediator (M), and the change score in strength endurance served as the dependent variable (Y). The significance of the relative indirect effects for each intervention was determined using bias-corrected bootstrapping with 5000 resamples. An indirect effect was considered statistically significant if its 95% confidence interval (CI) did not contain zero. This approach allowed us to examine the mechanistic pathway for the HRT and BFR-LRT interventions independently within a single, robust model.

## 3 Results

**Fig. 3:**
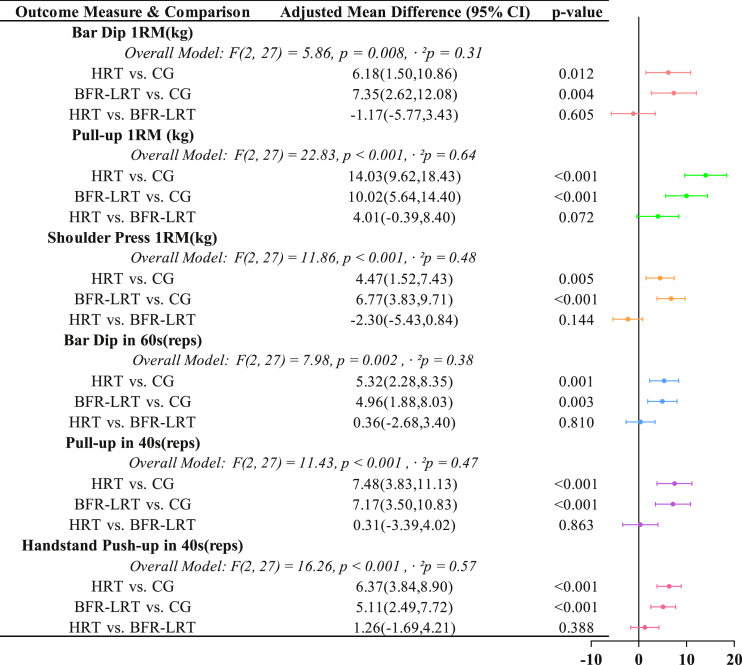
Adjusted Mean Differences in Post-Intervention Outcomes between the HRT, BFR-LRT, and CG groups. Abbreviations: CG, Control Group; HRT, High-Load Resistance Training; BFR-LRT, Blood Flow Restriction with Low-Load Training; CI, Confidence Interval. The *Overall Model* line presents the F-statistic with its between-groups and within-groups degrees of freedom, the exact p-value, and the partial eta-squared (η^2^p) effect size from the one-way ANCOVA. Partial eta-squared represents the proportion of variance in the dependent variable that is attributable to the group factor, after controlling for the effect of the baseline covariate. The ‘Adjusted Mean Difference' is the difference between group means adjusted for the baseline value of the outcome measure. An unadjusted alpha level of p < 0.05 was used to determine statistical significance for all pairwise comparisons.

The mean changes from pre-to post-intervention for all outcome measures are presented in Table 4, along with within-group effect sizes. The primary analysis, using ANCOVA to control for baseline scores, revealed a significant main effect of the intervention on all six outcome measures. The detailed results of these analyses, including overall model statistics and post-hoc pairwise comparisons, are presented in Fig. 3.

**Table 4:** The absolute changes from pre-to post-intervention and within-group effect sizes.

Outcome Measure & Group	Absolute Change (Mean ± SD)	Within-Group Effect Size (Cohen's d)
**Bar Dip 1RM(kg)**		
CG	0.67 ± 2.39	0.28
HRT	9.16 ± 6.75	1.36
BFR-LRT	10.92 ± 6.54	1.67
**Pull-up 1RM (kg)**		
CG	1.93 ± 3.78	0.51
HRT	15.11 ± 5.90	2.56
BFR-LRT	11.80 ± 6.32	1.87
**Shoulder Press 1RM(kg)**		
CG	2.71 ± 3.07	0.89
HRT	9.79 ± 4.65	2.11
BFR-LRT	7.13 ± 4.09	1.74
**Bar Dip in 60s(reps)**		
CG	1.90 ± 2.81	0.68
HRT	8.00 ± 3.68	2.17
BFR-LRT	8.60 ± 3.84	2.24
**Pull-up in 40s(reps)**		
CG	1.80 ± 3.55	0.51
HRT	8.40 ± 3.81	2.21
BFR-LRT	10.40 ± 3.92	2.65
**Handstand Push-up in 40s(reps)**		
CG	0.40 ± 1.78	0.23
HRT	4.70 ± 3.59	1.31
BFR-LRT	8.30 ± 3.74	2.22

Data are presented as Mean ± Standard Deviation (SD). The within-group effect size (Cohen's d) quantifies the magnitude of change from pre-to post-intervention for each group. Following current recommendations for analyzing paired data, it was calculated using the standard deviation of the change score (d = Mean_change_/SD_change_) to accurately reflect the magnitude of the change relative to the consistency of the intervention response. Abbreviations: CG, Control Group; HRT, High-Load Resistance Training; BFR-LRT, Blood Flow Restriction with Low-Load Training.

### 3.1 Upper-body maximal strength

Both training interventions led to significant improvements in all three measures of maximal strength compared to the control group. No significant difference was observed between the HRT and BFR-LRT groups.

#### 3.1.1 Bar dip 1RM

A significant overall effect was found for Bar Dip 1RM (F(2, 27) = 5.86, p = 0.008, η^2^p = 0.31). Post-hoc analysis revealed that both the HRT group (p = 0.012) and the BFR-LRT group (p = 0.004) demonstrated significantly greater improvements than the CG. No significant difference was observed between the HRT and BFR-LRT groups (p = 0.605; Adjusted mean differences: 1.17 kg [−5.77, 3.43]) (Fig. 3). To better quantify the evidence for this null finding, a Bayesian ANCOVA was performed. The analysis yielded a Bayes Factor of BF_01_ = 2.31, indicating that the observed data are approximately 2.3 times more likely under the null hypothesis of no difference than the alternative hypothesis. This provides anecdotal evidence supporting a comparable effect between the two training protocols.

#### 3.1.2 Pull-up 1RM

For Pull-up 1RM, the ANCOVA revealed a large and significant main effect (F(2, 27) = 22.83, p < 0.001, η^2^p = 0.64). Both the HRT and groups were significantly superior to the CG (both p < 0.001). The comparison between the HRT and BFR-LRT groups did not reach statistical significance (p = 0.072; Adjusted mean differences: 4.01 kg [−0.39, 8.40]), though a trend was observed in favor of the HRT group (Fig. 3). A corresponding Bayesian analysis produced a Bayes Factor of BF_01_ = 1.00, indicating the data were equally likely under the null and alternative hypotheses. This suggests the evidence is entirely equivocal, providing no support for either the presence or absence of a difference for this specific measure.

#### 3.1.3 Shoulder press 1RM

A significant main effect was also observed for Shoulder Press 1RM (F(2, 27) = 11.86, p < 0.001, η^2^p = 0.48). Post-hoc tests confirmed that both the HRT (p = 0.005) and BFR-LRT (p < 0.001) groups significantly increased their strength compared to the CG. No significant difference was detected between the two training interventions (p = 0.144; Adjusted mean differences: 2.30 kg [−5.43, 0.84]) (Fig. 3). The Bayesian analysis provided anecdotal evidence in favor of the null hypothesis (BF_01_ = 2.13).

### 3.2 Upper-body strength endurance

Substantial gains in all three measures of upper-body strength endurance were observed for both training interventions compared to the control condition. There was no significant difference between the HRT and BFR-LRT groups.

#### 3.2.1 Bar dips in 60s

The analysis revealed a significant effect of the intervention on the number of bar dips performed in 60 s (F(2, 27) = 7.98, p = 0.002, η^2^p = 0.38). Both the HRT (p = 0.001) and BFR-LRT (p = 0.003) groups had significantly higher adjusted mean repetitions than the CG. The comparison between the HRT and BFR-LRT groups was not statistically significant (p = 0.810; Adjusted mean differences: 0.36 reps [−2.68, 3.40]) (Fig. 3). Bayesian analysis provided anecdotal support for the null hypothesis (BF_01_ = 2.43).

#### 3.2.2 Pull-ups in 40s

A large, significant group effect was observed for pull-up repetitions (F(2, 27) = 11.43, p < 0.001, η^2^p = 0.47). Post-hoc analysis confirmed that the adjusted mean repetitions for both the HRT and BFR-LRT groups were significantly greater than for the CG (both p < 0.001). There was no significant difference between the HRT and BFR-LRT groups (p = 0.863; Adjusted mean differences: 0.31 reps [−3.39, 4.02]) (Fig. 3). The Bayesian analysis provided further anecdotal evidence for this finding (BF_01_ = 2.51).

#### 3.2.3 Handstand push-ups in 40s

Finally, a large and significant effect was found for handstand push-up performance (F(2, 27) = 16.26, p < 0.001, η^2^p = 0.57). Both the HRT (p < 0.001) and BFR-LRT (p < 0.001) groups performed significantly more repetitions than the CG after adjusting for baseline scores. Again, no significant difference was found between the two training interventions (p = 0.388; Adjusted mean differences: 1.26 reps [−1.69, 4.21]) (Fig. 3). The corresponding Bayes Factor (BF_01_ = 2.40) provided anecdotal evidence in favor of the null hypothesis.

### 3.3 Mediation of endurance gains by maximal strength

To address the possibility of training-specific mechanistic pathways, we conducted three separate mediation analyses, one for each exercise, to test whether the effect of each intervention (HRT vs. Control and BFR-LRT vs. Control) on strength endurance was independently mediated by gains in maximal strength. The full path coefficients for each model are presented in Table 5.

**Table 5:** Mediation analysis results for the effect of the intervention (X) on endurance gains (Y) via strength gains (M).

Exercise/Path	Comparison	Coefficient	*Std. Error*	95% Confidence Interval	p-value
**Handstand Push-up**					
a path (X → M)	HRT vs. CG	7.08	*1.78*	[3.42, 10.73]	<0.001
	BFR-LRT vs. CG	4.42	*1.78*	[0.76, 8.08]	0.020
b path (M → Y)	HRT	0.44	*0.21*	[0.00, 0.88]	0.048
	BFR-LRT	0.40	*0.24*	[-0.09, 0.90]	0.107
	CG	−0.13	*0.32*	[-0.79, 0.53]	0.697
c' path (Direct Effect)	HRT vs. CG	1.19	*1.99*	[-2.89, 5.27]	0.554
	BFR-LRT vs. CG	6.12	*1.69*	[2.66, 9.59]	0.001
**ab path (Indirect)**	**HRT vs. CG**	**3.11**	***1.998***	**[0.25, 7.41]**	-
	BFR-LRT vs. CG	1.78	*1.616*	[-0.32, 6.00]	**-**
**Pull-up**					
a path (X → M)	HRT vs. CG	13.19	*2.44*	[8.19, 18.19]	<0.001
	BFR-LRT vs. CG	9.87	*2.44*	[4.87, 14.87]	<0.001
b path (M → Y)	HRT	0.32	*0.21*	[-0.12, 0.75]	0.150
	BFR-LRT	0.04	*0.20*	[-0.37, 0.45]	0.852
	CG	0.31	*0.33*	[-0.37, 1.00]	0.353
c' path (Direct Effect)	HRT vs. CG	2.44	*3.26*	[-4.25, 9.13]	0.461
	BFR-LRT vs. CG	8.23	*2.58*	[2.95, 13.52]	0.004
ab path (Indirect)	HRT vs. CG	4.16	*3.334*	[-3.24, 9.52]	-
	BFR-LRT vs. CG	0.37	*3.609*	[-2.81, 4.72]	-
**Bar Dip**					
a path (X → M)	HRT vs. CG	8.49	*2.50*	[3.35, 13.63]	0.002
	BFR-LRT vs. CG	10.25	*2.50*	[5.11, 15.38]	<0.001
b path (M → Y)	HRT	0.13	*0.17*	[-0.21, 0.48]	0.432
	BFR-LRT	0.27	*0.17*	[-0.09, 0.63]	0.139
	CG	0.47	*0.48*	[-0.51, 1.45]	0.328
c' path (Direct Effect)	HRT vs. CG	4.96	*2.09*	[0.67, 9.25]	0.025
	BFR-LRT vs. CG	3.97	*2.35*	[-0.84, 8.79]	0.102
ab path (Indirect)	HRT vs. CG	1.14	*2.014*	[-2.58, 5.69]	-
	BFR-LRT vs. CG	2.73	*6.357*	[-1.38, 6.67]	-

All coefficients represent unstandardized values. M = Change in maximal strength; Y = Change in endurance repetitions. X comparisons are relative to the Control group. The a path represents the effect of the intervention on strength gains. The b path represents the conditional (simple slope) effect of strength gains on endurance gains within each group. The c' path represents the natural direct effect provided by PROCESS, appropriate for models including X × M interactions. The ab path is the indirect effect, considered significant if its 95% bootstrap CI does not contain zero. The significant indirect effect is highlighted in bold.

For handstand push-up performance, the analysis revealed a divergent mechanistic pathway. For the HRT group, the intervention led to significant gains in maximal shoulder press strength (a path: 7.08, p < 0.001), and these strength gains were significantly associated with improvements in endurance repetitions (b path: 0.44, p = 0.048). This resulted in a statistically significant indirect effect (ab path: 3.11, 95% CI [0.25, 7.41]), indicating that strength gain was a key mediator for the HRT intervention. For the BFR-LRT group, while the intervention also significantly increased maximal strength (a path: 4.42, p = 0.020), the subsequent relationship between strength gains and endurance was not significant (b path: 0.40, p = 0.107). Consequently, the indirect effect for BFR-LRT was not statistically significant (ab path: 1.78, 95% CI [−0.32, 6.00]).

For pull-up performance, both the HRT and BFR-LRT interventions produced large and significant gains in maximal strength (a paths: 13.19 and 9.87, respectively). However, for both groups, these strength gains were not significantly associated with gains in pull-up endurance (b paths were non-significant). As a result, the indirect effects were not statistically significant for either the HRT group (ab path: 4.16, 95% CI [−3.24, 9.52]) or the BFR-LRT group (ab path: 0.37, 95% CI [−2.81, 4.72]).

A similar pattern was observed for bar dip performance. Although both interventions were effective at increasing maximal dip strength (significant a paths), this increase in strength did not translate into a statistically significant association with bar dip endurance (non-significant b paths). Therefore, the indirect effects were not significant for either training modality.

## 4 Discussion

This randomized controlled trial was designed to compare the effects of low-load blood flow restriction training (BFR-LRT) against traditional high-load resistance training (HRT) and a control condition on upper-body maximal strength and strength endurance in elite male collegiate gymnasts. The principal finding of this study was twofold: first, both the BFR-LRT and HRT interventions were substantially more effective than regular gymnastics training alone for improving all six measures of upper-body performance. Second, and of greater clinical and practical importance, there were no statistically significant differences in the magnitude of improvements between the BFR-LRT and HRT groups. While a traditional p-value framework precludes formally accepting the null hypothesis, our follow-up Bayesian analyses allowed us to quantify the evidence for this finding. For five of the six outcome measures, we found anecdotal evidence (BF_01_ > 2.0) in favor of the null hypothesis of no difference. These results suggest that BFR-LRT, despite utilizing significantly lower mechanical loads, is a comparably effective training modality to traditional HRT for enhancing critical physical qualities in this highly specialized athletic population. This finding has profound practical implications for training periodization, load management, and injury risk mitigation in a sport characterized by extreme physical demands and high rates of overuse injury.

### 4.1 Interpretation of findings in the context of existing literature

The finding that BFR-LRT is as effective as HRT warrants careful consideration, as it both supports and extends the existing body of scientific literature. This conclusion of comparable efficacy is based not only on the absence of significant p-values, but also on the consistent, albeit anecdotal, Bayesian evidence favoring the null hypothesis. While numerous studies have confirmed that BFR-LRT is a potent stimulus for muscular adaptation, its efficacy relative to the gold standard of HRT has been a subject of ongoing investigation. Several comprehensive meta-analyses have concluded that while BFR-LRT is clearly superior to low-load training without BFR, traditional HRT generally remains superior for eliciting maximal strength gains, particularly in the lower body ^11^^,^^13^, ^14^, ^15^^,^^32^^,^^44^^,^^45^. Our results, which demonstrate a comparable effect between the two modalities with a degree of quantified evidence, therefore appear to diverge from this general consensus. This divergence is likely attributable to several key factors that differentiate the present study from much of the previous research, namely the training status of the participants, the exclusive focus on upper-body musculature, and the specific nature of the athletic population. Crucially, it must be acknowledged that the mid-intervention 1RM re-assessment in both training groups constituted a high-load training stimulus^46^, ^47^, ^48^. This repeated testing may have acted as a confounding variable, potentially contributing to the strength gains observed in the BFR-LRT group and narrowing the difference between the two training interventions.^48^

The training status of the participants is a critical factor in interpreting these results. The gymnasts in this study were elite athletes, characterized by years of high-volume, bodyweight-based training, placing them at an advanced stage of the adaptation continuum. However, a key distinction is that they were not concurrently engaged in a structured, high-load resistance training program. Therefore, both the BFR-LRT and HRT interventions represented a novel, supplementary training stimulus. The central challenge for such athletes is not a lack of training, but rather the management of the immense physical stress that already exists from over 15 h of weekly gymnastics practice. In this context, the findings suggest that for a highly trained population already subjected to significant mechanical and systemic stress, BFR-LRT is a comparably effective training modality to HRT. Its primary practical advantage is the ability to elicit significant strength gains with substantially lower mechanical loads, offering a valuable tool for coaches to add a potent adaptive stimulus while helping to mitigate cumulative joint stress and manage overall training volume.

It could be argued that a truly novel stimulus might be expected to produce superior, rather than comparable, results. One interpretation is that for this elite population, the novelty of the metabolic stimulus from BFR-LRT was sufficient to match the potent, albeit familiar, stimulus of HRT over a 6-week period, but not to exceed it. Furthermore, as our study was likely underpowered to detect smaller differences, we cannot rule out the possibility of a modest, yet meaningful, difference between the groups. Therefore, our results should be interpreted cautiously as demonstrating comparable effectiveness under the specific conditions of this trial, rather than proving true equivalence.

Furthermore, our study's focus on the multi-joint, compound upper-body movements, such as bar dips, pull-ups, and shoulder presses, which are highly specific to the demands of gymnastics, address a key question regarding the application of BFR. The primary mechanism is likely an indirect one, driven by compensatory motor unit recruitment. By applying occlusion to the arms, the biceps, brachialis, and triceps fatigue prematurely, becoming a “weak link” in the kinetic chain. To maintain force production and complete the movement, the central nervous system must compensate by increasing neural drive to the larger, un-occluded prime movers, such as the latissimus dorsi and deltoids.^49^^,^^50^ This effectively forces these proximal muscles to work at a higher intensity than the low external load would typically require. The functional carryover from these exercises may be different from that observed in studies using single-joint movements like bicep curls or leg extensions, potentially influencing the comparability of the outcomes. The study by Ozaki et al.,^29^ for instance, found a greater absolute strength increase in bench press with HIT compared to BFR- LRT. While our protocol shared similarities in duration and frequency, the use of gymnastics-specific exercises and a highly trained population likely contributed to our finding of comparable efficacy.

### 4.2 The movement- and modality-specific role of maximal strength in mediating endurance gains

In our initial discussion, we hypothesized that gains in strength endurance were primarily a consequence of improvements in maximal strength. Our follow-up mediation analysis, conducted separately for each training modality, reveals a more complex and nuanced reality. The findings indicate that the role of maximal strength as a statistical mediator is both movement-specific and dependent on the training intervention itself, strongly supporting the idea of divergent mechanistic pathways.

Our most compelling finding comes from the handstand push-up analysis. For the HRT group, the results align perfectly with the traditional strength training paradigm: the intervention significantly increased maximal strength (a strong ‘a' path), and this gain in strength was directly and significantly associated with an increase in endurance repetitions (a significant ‘b' path). This confirms a significant indirect effect, showing that HRT improved handstand push-up endurance largely by making the athletes stronger. In stark contrast, this mediating pathway was absent for the BFR-LRT group. While BFR-LRT also effectively increased maximal strength, this strength gain did not have a statistically significant relationship with the improvement in endurance (a non-significant ‘b' path). This suggests that the endurance benefits of BFR-LRT in this complex pushing movement stem from other, non-load-dependent adaptations. Plausible mechanisms include enhanced local muscular fatigue resistance, improved metabolic efficiency from training in a hypoxic environment, or other neuromuscular adaptations not fully captured by a 1RM assessment. Our result is consistent with recent work that has also demonstrated, via mediation analysis, that strength increases are a direct statistical mediator of improvements in absolute endurance.^51^

Interestingly, for both the pull-up and bar dip movements, we did not find a significant indirect effect for either training group. It is crucial to interpret this finding carefully. In all cases, both HRT and BFR-LRT produced substantial and significant gains in maximal strength (strong ‘a' paths). The lack of a significant mediation effect was driven by a non-significant ‘b' path, meaning the magnitude of strength gain did not statistically predict the magnitude of endurance gain within our model. This may be attributable to the fact that, for these fundamental and highly practiced gymnastics movements, endurance is a multifaceted construct influenced by factors beyond prime-mover strength alone. Adaptations in stabilizing muscles, enhanced inter-muscular coordination, and technical proficiency under fatigue may also play significant roles, and these adaptations are not necessarily reflected in a 1RM test.

In summary, our refined analysis moves beyond the initial hypothesis. For the handstand push-up, our data clearly shows that HRT works through strength gains, while BFR-LRT works through other mechanisms. For other movements, the relationship is less direct, suggesting that while strength is essential, it is part of a larger constellation of adaptations that drive improvements in muscular endurance.

### 4.3 Practical applications and implications for gymnastics

The findings of this study have substantial practical implications for coaches, athletes, and sports medicine practitioners working with gymnasts. The demonstration that BFR-LRT is a viable alternative to HRT provides a valuable new tool that can be strategically integrated into an athlete's training plan to optimize performance while mitigating risk. First and foremost, BFR-LRT can serve as a critical tool for load management and injury risk reduction. Gymnastics has one of the highest injury rates among all sports, with the upper extremities being particularly vulnerable to overuse injuries due to the extreme and repetitive loading on the shoulder, elbow, and wrist joints.^4^ By allowing for significant strength gains with mechanical loads as low as 30% 1RM, BFR-LRT can be used to maintain or even enhance strength and endurance while substantially deloading these vulnerable joints. This could be strategically implemented during periods of high-volume skill training or in athletes with a history of joint pain. Second, BFR-LRT can be intelligently integrated into a periodized training program. During intense competitive phases, where recovery is paramount and systemic fatigue from heavy lifting could interfere with technical performance, BFR-LRT can serve as a less taxing yet still potent stimulus to maintain strength. Conversely, it could be used as a supplementary tool during the preparatory phase to add training volume without a commensurate increase in mechanical wear and tear.^52^ Finally, BFR-LRT has significant potential in rehabilitation settings. Following an injury, when heavy loading is contraindicated, athletes often struggle with significant muscle atrophy and strength loss. BFR-LRT provides a safe and effective means to stimulate muscle hypertrophy and strength early in the rehabilitation process, potentially accelerating an athlete's return to sport.^15^^,^^32^^,^^53^

### 4.4 Strengths and limitations

This study possesses several methodological strengths that enhance the credibility of its findings. The randomized, controlled design and adherence to CONSORT guidelines provide a robust framework for causal inference. The use of a highly specific, elite athletic population addresses a critical gap in the existing literature. Furthermore, the use of ANCOVA to control for baseline scores represents a rigorous statistical approach that increases the validity of the between-group comparisons, while the preliminary reliability testing of our sport-specific outcome measures ensures that our data are both valid and consistent.

Despite these strengths, the study's limitations must be acknowledged. A primary limitation is the relatively small sample size (n = 10 per group). A sensitivity analysis indicated that the study was underpowered to reliably detect smaller, yet potentially meaningful, differences between the HRT and BFR-LRT groups. This increases the risk of Type II errors, where a true effect may be missed.^54^ To address this inherent limitation and more formally interpret our null findings, we supplemented our analysis with a Bayesian framework. This approach allowed us to move beyond simply failing to reject the null hypothesis and instead quantify the evidence in its favor. The results consistently provided anecdotal evidence for the null hypothesis (BF_01_ > 2.0 for five of six outcomes), with one outcome showing equivocal evidence (BF_01_ = 1.00). While this level of evidence does not prove true equivalence, it provides a more informative interpretation than a p-value alone. It suggests that any true difference between the interventions, if one exists, is likely smaller than our study could detect and that the data are more consistent with a model of comparable effects. The wide confidence intervals surrounding the between-group comparisons reflect this remaining uncertainty, and clinically relevant differences in favor of one modality over the other cannot be definitively excluded.

A second substantial limitation involves the potential for confounding variables introduced by the study's own assessment protocol. The mid-point 1-repetition maximum (1RM) assessment, while necessary for tracking progress, functioned as an unplanned high-load training stimulus for the intervention participants.^48^ This event could have obscured the true effects of the low-load BFR intervention, effectively creating a hybrid training model. However, the study's robust design, which features a non-exercise control group, provides a crucial advantage for interpretation. As highlighted by Hammert et al. (2024),^31^ such a control is a major methodological strength, essential for isolating the intervention's effects from external factors and allowing for a more confident attribution of the observed outcomes to the BFR training itself.

Further methodological limitations should be considered. First, the generalizability of these findings is limited to the specific population studied: young, male, elite collegiate gymnasts. Second, the use of different cuffs for measuring and applying BFR may have introduced additional variability in the BFR group's responses. Applying 70% SBP with a 6 cm cuff could have resulted in supra-occlusive pressure for some participants but not others, adding a potential source of variance. Additionally, while our preliminary testing established the acute reliability of our sport-specific outcome measures, this 48-h assessment has limited value for quantifying measurement error over the full 6-week intervention. However, this limitation is substantially mitigated by the inclusion of a non-exercise control group, which provides a practical and more valid estimate of long-term measurement variability,^31^ thereby strengthening the interpretation of the changes observed in the training groups. A final methodological nuance is the inclusion of the reliability testing sample within the main trial's participant pool. This meant a subset of participants (n = 20) had additional exposure to the testing protocol before the intervention began, which could have introduced additional training stimulus. While randomization after preliminary testing likely prevented systematic bias, it may have increased overall variability within each group, potentially reducing our statistical power to detect true between-group differences.

Finally, limitations exist regarding the study's duration and outcome measures. The 6-week intervention, while sufficient to elicit initial adaptations, cannot provide insight into the long-term sustainability of these gains. Furthermore, the muscular endurance metrics used in this study—while validated for reliability—are specialized performance tests highly specific to the demands of gymnastics. It remains to be confirmed whether these adaptations would translate directly to more traditional measures of local muscular endurance, such as repetitions to failure at a given percentage of 1RM. As discussed in a recent review by Hammert et al. (2025),^55^ this distinction is important when contextualizing findings related to “absolute” versus “relative” endurance. This study also did not include direct mechanistic measures, such as muscle cross-sectional area via ultrasound or neural activation via electromyography (EMG).

### 4.5 Future research directions

The findings and limitations of this study provide a clear roadmap for future research. The most pressing need is for a larger, adequately powered multi-center randomized controlled trial to definitively determine the relative efficacy of BFR-LRT and HRT in this population. Such a study would provide a more precise estimate of the effect sizes and could confirm whether the two modalities are truly comparable or if subtle differences exist.

Longitudinal studies are also needed to investigate the effects of integrating BFR-LRT over an entire competitive season. This would provide invaluable data on long-term adaptations, the potential for non-responders, and the chronic effects on injury rates and performance. Furthermore, future investigations should incorporate direct mechanistic measures, such as ultrasound imaging to track muscle hypertrophy and EMG to assess changes in neural drive, to elucidate the precise physiological pathways driving the adaptations observed in elite gymnasts. Finally, research is critically needed to expand this line of inquiry to elite female gymnasts, who represent a large but understudied population with unique physiological characteristics and injury profiles.

## 5 Conclusion

In this cohort of elite gymnasts, a 6-week BFR-LRT program produced comparable adaptations in upper-body strength and strength endurance to traditional HRT, with Bayesian analysis providing evidence to support this comparable efficacy. Of significant mechanistic importance, our findings also reveal that while the performance outcomes were similar, the underlying physiological pathways may be both movement- and modality-specific. For the handstand push-up, endurance gains in the HRT group were statistically mediated by increases in maximal strength. In contrast, this pathway was not significant for the BFR-LRT group, suggesting it enhances endurance through alternative, non-load-dependent mechanisms. These findings provide strong evidence that BFR-LRT is a viable training alternative that can elicit significant strength gains with lower mechanical loads. For coaches and athletes, this offers a valuable, evidence-based tool to manage training volume and mitigate joint stress, potentially by stimulating a different constellation of physiological adaptations than traditional heavy training.

## Author contributions

Aojie Li was instrumental in the conceptualization, methodology, investigation, data curation, formal analysis, and initial writing of the original draft. Jing Tang and Kaiqi Zheng both contributed to the investigation, with Jing Tang also handling data curation and both contributing to writing – review & editing. Jingyi Chen focused on investigation, visualization, and writing – review & editing. Guangshun Wang provided essential resources, alongside his contributions to writing – review & editing. Daoguang Feng played a crucial role in the conceptualization, project administration, supervision, and funding acquisition, as well as writing – review & editing. All authors agree to be accountable for the content of the work.

## Role of the funding source

This research was supported by the “Outstanding Postgraduate Overseas Joint Training Programme” of 10.13039/501100003169South China Normal University.

## Declaration of interests

The authors have no conflicts of interest relevant to this article.

## References

[1] Huschtscha Z., Silver J., Gerhardy M. (2024). The effect of palmitoylethanolamide (PEA) on skeletal muscle hypertrophy, strength, and power in response to resistance training in healthy active adults: a double-blind randomized control trial. Sports Med - Open.

[2] Flewwelling L.D., Hannaian S.J., Cao V. (2025). What are the potential mechanisms of fatigue-induced skeletal muscle hypertrophy with low-load resistance exercise training?. Am J Physiol Cell Physiol.

[3] Lilja M., Moberg M., Apró W. (2023). Limited effect of over-the-counter doses of ibuprofen on mechanisms regulating muscle hypertrophy during resistance training in young adults. J Appl Physiol.

[4] Edouard P., Steffen K., Junge A. (2018). Gymnastics injury incidence during the 2008, 2012 and 2016 olympic games: analysis of prospectively collected surveillance data from 963 registered gymnasts during olympic games. Br J Sports Med.

[5] Gabbett T.J. (2016). The training—injury prevention paradox: should athletes be training smarter and harder?. Br J Sports Med.

[6] Issurin V.B. (2010). New horizons for the methodology and physiology of training periodization.

[7] Göpfert B., Schärer C., Tacchelli L. (2022). Frequency shifts in muscle activation during static strength elements on the rings before and after an eccentric training intervention in Male gymnasts. J Funct Morphol Kinesiol.

[8] Paunović M., Đorđević D., Marinković D. (2023). Is the handgrip strength influential factor on the competition result in elite male artistic gymnasts?.

[9] Buckner S.L., Jessee M.B., Dankel S.J. (2020). Blood flow restriction does not augment low force contractions taken to or near task failure. Eur J Sport Sci.

[10] Jessee M.B., Buckner S.L., Mouser J.G. (2018). Muscle adaptations to high-load training and very low-load training with and without blood flow restriction. Front Physiol.

[11] Lixandrão M.E., Ugrinowitsch C., Berton R. (2018). Magnitude of muscle strength and mass adaptations between high-load resistance training versus low-load resistance training associated with blood-flow restriction: a systematic review and meta-analysis. Sports Med.

[12] Mouser J.G., Ade C.J., Black C.D. (2018). Brachial blood flow under relative levels of blood flow restriction is decreased in a nonlinear fashion. Clin Physiol Funct Imag.

[13] Grønfeldt B.M., Lindberg Nielsen J., Mieritz R.M. (2020). Effect of blood‐flow restricted vs heavy‐load strength training on muscle strength: systematic review and meta‐analysis. Scand J Med Sci Sports.

[14] Wortman R.J., Brown S.M., Savage-Elliott I. (2021). Blood flow restriction training for athletes: a systematic review. Am J Sports Med.

[15] Scott B.R., Loenneke J.P., Slattery K.M. (2016). Blood flow restricted exercise for athletes: a review of available evidence. J Sci Med Sport.

[16] Loenneke J.P. (2021). Muscle growth does not contribute to the increases in strength that occur after resistance training. Med Sci Sports Exerc.

[17] Škarabot J., Brownstein C.G., Casolo A. (2021). The knowns and unknowns of neural adaptations to resistance training. Eur J Appl Physiol.

[18] Hortobágyi T., Granacher U., Fernandez-del-Olmo M. (2021). Functional relevance of resistance training-induced neuroplasticity in health and disease. Neurosci Biobehav Rev.

[19] Bennett H., Slattery F. (2019). Effects of blood flow restriction training on aerobic capacity and performance: a systematic review. J Strength Condit Res.

[20] Yin M., Deng S., Deng J. (2025). Physiological adaptations and performance enhancement with combined blood flow restricted and interval training: a systematic review with meta-analysis. J Sport Health Sci.

[21] Geng Y., Wu X., Zhang Y. (2024). Potential moderators of the effects of blood flow restriction training on muscle strength and hypertrophy: a meta-analysis based on a comparison with high-load resistance training. Sports Med - Open.

[22] Su Y., Wang F., Wang M. (2024). Effects of blood flow restriction training on muscle fitness and cardiovascular risk of obese college students. Front Physiol.

[23] Schärer C., Tacchelli L., Göpfert B. (2019). Specific eccentric–isokinetic cluster training improves static strength elements on rings for elite gymnasts. Int J Environ Res Publ Health.

[24] Schärer C., Bucher P., Lüthy F. (2022). Combined eccentric-isokinetic and isoinertial training leads to large ring-specific strength gains in elite gymnasts. Sports.

[25] Lockard M.M., Gable T.F. (2018). Implementing a progressive resistance training program in youth junior olympic women's gymnastics: 2190 board #26 June 1 9: 30 AM - 11: 00 AM. Med Sci Sports Exerc.

[26] Karagianni K., Donti O., Bogdanis G.C. (2019). Effects of a supplementary strength-power training program on neuromuscular performance in young female athletes. Proceedings.

[27] Moher D., Schulz K.F., Altman D.G. (2001). The CONSORT statement: revised recommendations for improving the quality of reports of parallel group randomized trials. BMC Med Res Methodol.

[28] Moher D., Schulz K.F., Altman D. (2001). The CONSORT statement: revised recommendations for improving the quality of reports of parallel-group randomized trials. JAMA.

[29] Ozaki H., Yasuda T., Ogasawara R. (2013). Effects of high-intensity and blood flow-restricted low-intensity resistance training on carotid arterial compliance: role of blood pressure during training sessions. Eur J Appl Physiol.

[30] Hammert W.B., Kataoka R., Yamada Y. (2024). Progression of total training volume in resistance training studies and its application to skeletal muscle growth. Physiol Meas.

[31] Hammert W.B., Dankel S.J., Kataoka R. (2024). Methodological considerations when studying resistance-trained populations: ideas for using control groups. J Strength Condit Res.

[32] Scott B.R., Loenneke J.P., Slattery K.M. (2015). Exercise with blood flow restriction: an updated evidence-based approach for enhanced muscular development. Sports Med.

[33] Zheng X., Yuan W., Chen Y. (2014). Research on physical fitness evaluation standard and model construction of sports colleges' gymnastics major student: based on factor analysis. Journal of Beijing Sport University.

[34] Johnson D., Lynch J., Nash K. (2009). Relationship of lat-pull repetitions and pull-ups to maximal lat-pull and pull-up strength in men and women. J Strength Condit Res.

[35] Koo T.K., Li M.Y. (2016). A guideline of selecting and reporting intraclass correlation coefficients for reliability research. J Chiropract Med.

[36] Atkinson G., Nevill A.M. (1998). Statistical methods for assessing measurement error (Reliability) in variables relevant to sports medicine. Sports Med.

[37] Vickers A.J., Altman D.G. (2001). Analysing controlled trials with baseline and follow up measurements. Bmj.

[38] Rubin M. (2024). Inconsistent multiple testing corrections: the fallacy of using family-based error rates to make inferences about individual hypotheses. Methods Psychol.

[39] Cohen J. (2013).

[40] Dankel S.J., Loenneke J.P. (2021). Effect sizes for paired data should use the change score variability rather than the pre-test variability. J Strength Condit Res.

[41] Dankel S.J., Mouser J.G., Mattocks K.T. (2017). The widespread misuse of effect sizes. J Sci Med Sport.

[42] Sedgwick P. (2015). Randomised controlled trials: understanding effect sizes. BMJ.

[43] Hayes A.F. (2017). A Regression-based Approach.

[44] Slysz J., Stultz J., Burr J.F. (2016). The efficacy of blood flow restricted exercise: a systematic review & meta-analysis. J Sci Med Sport.

[45] Pignanelli C., Christiansen D., Burr J.F. (2021). Blood flow restriction training and the high-performance athlete: science to application. J Appl Physiol.

[46] Dankel S.J., Counts B.R., Barnett B.E. (2017). Muscle adaptations following 21 consecutive days of strength test familiarization compared with traditional training: 1RM training. Muscle Nerve.

[47] Mattocks K.T., Buckner S.L., Jessee M.B. (2017). Practicing the test produces strength equivalent to higher volume training. Med Sci Sports Exerc.

[48] Spitz R.W., Bell Z.W., Wong V. (2020). Strength testing or strength training: considerations for future research. Physiol Meas.

[49] Yasuda T., Fujita T., Miyagi Y. (2006). Electromyographic responses of arm and chest muscle during bench press exercise with and without KAATSU. Int J KAATSU Train Res.

[50] Yasuda T., Fujita S., Ogasawara R. (2010). Effects of low-intensity bench press training with restricted arm muscle blood flow on chest muscle hypertrophy: a pilot study. Clin Physiol Funct Imag.

[51] Chatlaong MA, Mouser JG, Bentley JP, et al. Mechanisms Mediating Increased Endurance Following high- and low-load Training with and Without Blood Flow Restriction n.d.

[52] Smith H.K., Bird S.P., Coskun B. (2025). Effectiveness of blood flow restriction training during a taper phase in basketball players. J Sports Sci.

[53] Hughes L., Paton B., Rosenblatt B. (2017). Blood flow restriction training in clinical musculoskeletal rehabilitation: a systematic review and meta-analysis. Br J Sports Med.

[54] Button K.S., Ioannidis J.P.A., Mokrysz C. (2013). Power failure: why small sample size undermines the reliability of neuroscience. Nat Rev Neurosci.

[55] Hammert W.B., Yamada Y., Kataoka R. (2025). Changes in absolute and relative muscular endurance after resistance training: a review of the literature with considerations for future research. J Strength Condit Res.

